# Medical Efforts and Injury Patterns of Military Hospital Patients Following the 2013 Lushan Earthquake in China: A Retrospective Study

**DOI:** 10.3390/ijerph120910723

**Published:** 2015-08-31

**Authors:** Peng Kang, Bihan Tang, Yuan Liu, Xu Liu, Zhipeng Liu, Yipeng Lv, Lulu Zhang

**Affiliations:** Institute of Military Health Management, Second Military Medical University, 800 Xiangyin Rd, 200433, Shanghai, China; E-Mails: kpkp315@163.com (P.K.); mangotangbihan@126.com (B.T.); yawnlau@126.com (Y.L.); xuliuaqua@126.com (X.L.); liuzhipeng_1987@sina.com (Z.L.); epengl@163.com (Y.L.);

**Keywords:** injury, injury patterns, rescue management, military hospital, Lushan, earthquake injuries, natural disaster, medical evacuation, trauma

## Abstract

The aim of this paper is to investigate medical efforts and injury profiles of victims of the Lushan earthquake admitted to three military hospitals. This study retrospectively investigated the clinical records of 266 admitted patients evacuated from the Lushan earthquake area. The 2005 version of the Abbreviated Injury Scale (AIS-2005) was used to identify the severity of each injury. Patient demographic data, complaints, diagnoses, injury types, prognosis, means of transportation, and cause of injury were all reviewed individually. The statistical analysis of the study was conducted primarily using descriptive statistics. Of the 266 patients, 213 (80.1%) were admitted in the first two days. A total of 521 injury diagnoses were recorded in 266 patients. Earthquake-related injuries were primarily caused by buildings collapsing (38.4%) and victims being struck by objects (33.8%); the most frequently injured anatomic sites were the lower extremities and pelvis (34.2%) and surface area of the body (17.9%). Fracture (41.5%) was the most frequent injury, followed by soft tissue injury (27.5%), but crush syndrome was relatively low (1.2%) due to the special housing structures in the Lushan area. The most commonly used procedure was suture and dressings (33.7%), followed by open reduction and internal fixation (21.9%).The results of this study help formulate recommendations to improve future disaster relief and emergency planning in remote, isolated, and rural regions of developing countries.

## 1. Introduction

**Figure 1:**
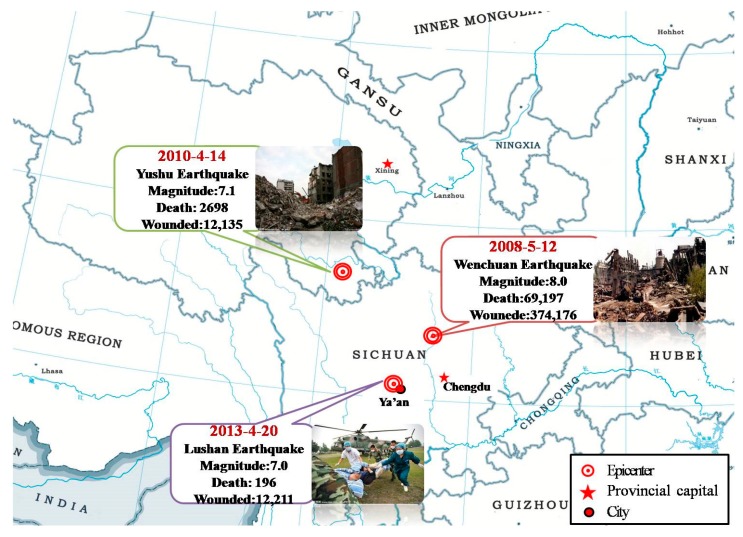
Overview of three catastrophic earthquakes in the past ten years in China.

Compared with other types of natural disasters, earthquakes are much more harmful and unpredictable, causing dramatic casualties and a significant loss of assets [1,2]. Western China has been identified as an earthquake-prone area, especially in the past decade, because a series of catastrophic earthquakes have occurred in succession, including the 2008 Wenchuan earthquake (magnitude (Mw): 8.0) and the 2010 Yushu earthquake (Mw: 7.1), and the 2013 Lushan earthquake (Mw: 7.0) which, combined, had a total of 72,091 reported deaths and left 398,522 injured (Figure 1) [3]. These earthquakes have brought disaster preparedness to the forefront of national policy of China. In order to allocate medical resources and implement medical relief better, it is necessary to know the characteristics of injuries sustained in earthquakes.

The injuries caused by earthquakes were always highly mechanical and often multisystem, requiring intensive curative medical and surgical care at a time when the local and regional medical response capacities have been at least partly disrupted, which presented sudden and serious crises for health management and treatment centers [4]. Many studies have focused on the injury patterns of earthquake victims. Doocy *et al*. also made a systematic literature review on the human impact of earthquakes and found that soft tissue injuries (including lacerations and contusions) and fractures were the most common types of injury reported, and the extremities were the most likely areas of the body to be affected [5]. Some studies also focused on multiple injuries since obtaining more and better information about the types and profiles of these multiple injuries can add essential information to better plan and more readily adapt the surgical management of the injured following earthquakes [6].

Overall there is a similar pattern of injuries across a wide range of earthquakes, which allows for a certain prediction of types of injuries and the type of treatment needed. However, variations of injury pattern still exist depending on the severity of seismic activity and other factors. There are still substantial challenges in estimating the exact burden of earthquake injuries because of variation in classification and reporting procedures, underreporting due to a lack of data collection, and a failure to aggregate and maintain centralized reporting in emergency contexts [7]. Although many earthquake victims suffer injuries requiring surgical intervention, major gaps in knowledge on the epidemiology of an earthquake compromise the surgical delivery. What is the exact injury burden and distribution of procedures performed after major earthquakes? More and better information about the types and profiles of these earthquake-related injuries are needed.

At 8:02 am on 20 April 2013, a 7.0 Mw earthquake hit the Lushan County in Sichuan province, a precipitous, mountainous region in western China, approximately 100 kilometers from Chengdu City. The landscape proved to be a challenge for medical rescuers and caused considerable strain on China’s emergency medical system due to a lack of medical resources. The widespread effects of the earthquake caused more than 2.3 million people to lose their homes within the most seriously devastated area, which spanned15720 km^2^; the earthquake ultimately claimed the lives of 196 victims and more than 12,000 were officially reported as injured [8].

To the best of our knowledge, this paper is one of the first to investigate the medical efforts and injury profiles of victims of the Lushan earthquake in depth. The aim is to investigate the injury profiles in patients admitted to three military hospitals following the Lushan earthquake, with a special focus on the injury patterns, subsequent treatment, and rescue management; this will help formulate recommendations to improve future disaster relief and emergency planning in remote, isolated, rural regions of developing counties.

## 2. Materials and Methods

### 2.1. Study Design

This study retrospectively investigated the clinical records of admitted patients evacuated from the Lushan earthquake area since 20 April 2013. All three military hospitals that admitted patients from the disaster area participated in the present study. Of these, one is located in Ya’an City, only 10 km from the epicenter of the Lushan earthquake; this facility serves as a “forward hospital” within the quake area to admit those with earthquake-related injuries. The other two were located in Chengdu City; these were “peripheral hospitals” with the purpose of relieving the medical care pressure and securing as many inpatient beds as possible to preserve the resource availability in forward hospitals. To obtain detailed information about the management of medical relief work, we also held interviews with commanders of the hospital medical professionals and members of medical rescue teams.

### 2.2. Data Collection

Our survey was performed between 7 August 2013 and 17 August 2013. Five researchers were dispatched to Sichuan province for data collection in the three military hospitals. We reviewed and collected the clinical records of 298 earthquake-related patients who were admitted to the surveyed hospitals from 20 April 2013 to 20 May 2013, based on the medical records database. After excluding 32 due to missing data for key variables or contact information (e.g., age, 10 patients; diagnoses, 16 patients; contact information, six patients), 266 eligible patients were included in this study.

### 2.3. Key Variables

Patient demographic data and variables including phone number, home address, complaints, diagnoses, injury types, disposition (including admission, discharge, and transfer), prognosis, rescuer (yes/no), death (yes/no), disabled (yes/no), and means of transportation were all individually reviewed. All diagnoses for patients with injuries or diseases were based on the final hospital diagnosis according to the attending physician. To ascertain the cause of every injury, interviews were conducted with every patient or his/her relative by telephone, using the phone numbers on record by consulting the medical record database. The telephone investigation was conducted by five professional researchers, whereby a complete description of the survey was presented to the study candidates, followed by the procurement of informed verbal consent, each participant independently responded to the questions and recalled the cause of the injury. If this patient was unavailable for the survey, their household member who could remember the whole traumatic process during the earthquake and describe the cause of the injury in detail was also selected. If the surveyed participant was not available after two contact attempts, he or she would be omitted and removed from the survey.

### 2.4. Injury Coding

The injured sites were categorized based on the body region affected (head, neck, face, chest, spine, abdomen/internal organs, upper extremities, lower extremities/pelvis, and body surface/others) and the severity of each injury was identified based on the 2005 version of the Abbreviated Injury Scale (AIS-2005). The AIS is a comprehensive taxonomy of individual injuries that denotes body region, type of anatomic structure, nature, and severity of injury. The severity index ranges from 0 (*no injury*) to 6 (*fatal injury*) [9,10,11].

### 2.5. Statistical Analysis

For statistical analysis, descriptive statistics were mainly used throughout the study. Categorical variables are presented as frequencies and percentages, and continuous variables are presented as mean T standard deviations. All data were analyzed using standard statistical software (SAS system, version 8.2, SAS Institute Inc., Cary, NC, USA).

### 2.6. Ethical Considerations

This study was approved by all of the participating hospitals and the Ethical Committee of the Second Military Medical University. No informed consent was necessary as this study used existing data.

## 3. Results

### 3.1. Medical Evacuation and Admission

**Figure 2:**
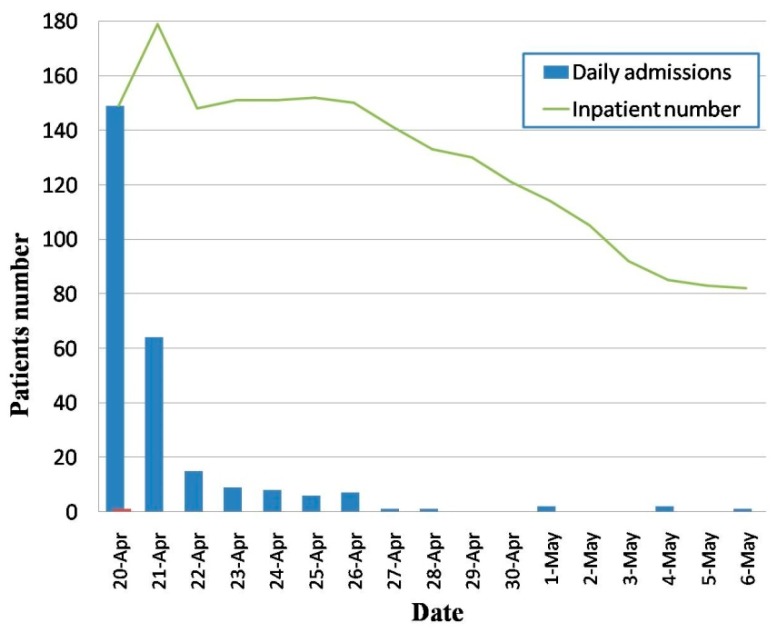
Distribution of the patients admitted to the three military hospitals on the basis of their date of admission and discharge.

After the Lushan earthquake, local medical institutions and peripheral rescue teams from the army and all over the nation launched a contingency plan in the initial hours of the disaster and took action to begin rescue operations immediately. Due to the lack of medical resources in the Lushan earthquake area, there was an immediate decision to medically evacuate the wounded and this process began on the first day of the earthquake because of the experience gained in the Yushu and Wenchuan earthquakes. Land evacuation played a key role in transporting the injured directly to the hospitals in Ya’an and Chengdu. Ambulances, buses, and even private cars were the primary source of transportation for most of the injured. Several helicopters from the Chinese Air Force were also used for transporting the severely wounded to the military hospitals in Chengdu. Of the 266 patients in this study, 56.0% (*n* = 149) were admitted on the first day of the disaster and 80.1% (*n* = 213) were admitted in the first two days. Sixty patients (22.6%) were transported by helicopters. Due to this situation, the number of hospitalized patients (the number of discharged patients were subtracted) reached a peak in the second day after the earthquake, but decreased steeply on the next day and subsequently began to dwindle. Dynamic variation of admissions and hospitalization of the 266 patients is shown in Figure 2.

### 3.2. General Injury Profile

**Figure 3:**
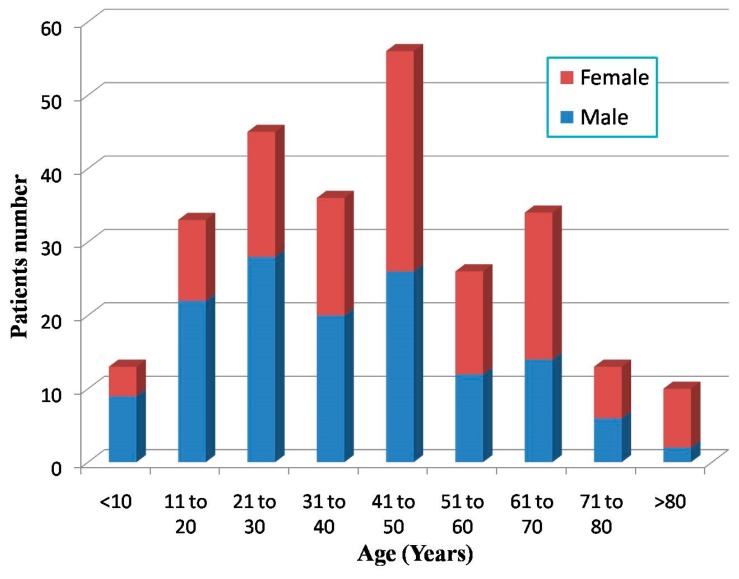
Age and gender distribution of the earthquake-related patients admitted to the three military hospitals.

The mean age of all hospitalized patients was 40.01 ± 20.58 years. Of these, 139 (52.1%) were male with a median age of 37 years; 127 (47.7%) were female with a median age of 46 years (range: 0 to 89 years). Age distribution for hospitalized patients is shown in Figure 3. Most patients (118, 44.4%) were middle-aged (31–50 years). Two males and eight females were older than 80 years. Generally, the male patients were significantly younger than the female ones.

Table 1 shows the number of injury diagnoses in admitted patients with earthquake-related injuries. Of the 266 admitted patients in the present study, a higher percentage of patients presented multiple injuries (55.7%) rather than with single injuries (44.4%). Among the patients with multiple injuries, patients presenting with two injuries were the prominent profile of this group (27.4%). Patients diagnosed with three injuries accounted for 16.9%, followed by ones with more than three injury diagnosis (11.3%).

**Table 1:** Number of injury diagnoses in admitted patients with earthquake-related injuries.

Number of Injury Diagnoses	Number (%)
1	118 (44.4)
2	73 (27.4)
3	45 (16.9)
>3	30 (11.3)

A total of 521 injury diagnoses were recorded in 266 patients (Table 2). A large share of these injuries was factures (41.5%), followed by soft tissue injury (27.5%), and contusions and lacerations (25.0%). Closed injuries were diagnosed in 12 patients (4.5%). Crush injuries and burn injuries were rare (1.2% and 1.0%, respectively). By body region, lower extremities, including the pelvis, and body surface had a large part of the injury burden, accounting for 52.1% (see Table 2).

Table 2 also presents the most relevant injury diagnoses by body region. Contusion, which includes lacerations, was the most frequent head-injury diagnosis (78.1%), followed by soft tissue injury (9.6%) and fractures (8.2%). Fractures were the most important diagnosed injury in the spine (96.2%). Fractures were also the predominant diagnosis in most of the remaining body regions: lower extremities and pelvis (57.3%), upper extremities (43.8%), thorax (55.6%), and face (39.2%). However, people were fortunate in the neck and abdominal regions. Only four (1.5%) injuries occurred in the abdomen and none in the neck.

**Table 2:** Diagnoses by body region of 521 injury diagnoses in admitted patients with earthquake-related injuries.

Injury Diagnoses	Body Region								Total Number (%)
	Head	Face	Thorax	Spine	Upper Extremities	Lower Extremities and Pelvis	Abdomen	Body Surface and Others	
Fracture	7	11	25	50	21	102	0	0	216 (41.5)
Soft tissue injury	6	3	2	1	11	29	1	90	143 (27.5)
Contusion & Laceration	57	13	10	0	13	33	2	2	130 (25.0)
Crush injury	0	0	1	0	1	4	0	0	6 (1.2)
Burns and Scalds	0	0	0	0	0	4	0	1	5 (1.0)
Closed injury	3	1	7	0	0	0	1	0	12 (2.3)
Dislocation	0	0	0	1	2	6	0	0	9 (1.7)
Total Number (%)	73 (14.0)	28 (5.4)	45 (8.6)	52 (10.0)	48 (9.2)	178 (34.2)	4 (0.8)	93 (17.9)	521 (100)

The data presented in Table 2 also show the distribution of each injury diagnosis across body regions. The largest proportions of fractures occurred in the lower extremities and pelvis (47.2%) and spine (23.1%). Almost all soft tissue injuries were identified at the body surface and other areas (96.8%). Contusions and lacerations were most diagnosed in the head (43.8%). Most closed injuries were in the thorax (58.3%). Crush injury, dislocation, and burns were rare but clustered in the lower extremities and pelvis (66.7%, 80.0%, and 66.7%, respectively).

Table 3 shows the cause of each injury diagnosis across body region. Hit/trapped by building and hit/trapped by objects were the top two causes of injury in most body regions. Hit/trapped by building (38.4%) accounted for the largest proportion and the most common region was the lower extremities and pelvis (43.5%), followed by the spine and body surface, accounting for 12.5% and 13.0%, respectively. Lower extremities and pelvis (25.0%), body surface (25.0%), and head (22.2%) were the top three high-risk injury regions for the cause, hit/trapped by objects. Seventy-three (14.0%) injury diagnoses were caused by falling, most of which occurred in the lower extremities and pelvis (32.9%), upper extremities (15.1%), and spine (15.1%). Due to cutting/piercing, almost all injuries were identified in the upper extremities (84.6%, 11/13).

In Table 4, the severity of each body injury was evaluated using theAIS-2005. The scores of 1 (*minor*) to 6 (*fatal*) were assigned to each body region. Of all the injuries, injuries with AIS scores 1–2, 3, and 4 accounted for 86.1%, 12.5%, and 1.2%, respectively. No one suffered from a fatal injury. There were five severe head injuries with an AIS score > 4 accounting for 6.8% of all the head injuries. Other injured regions with an AIS > 4 were the spine (5.8%) and lower extremities and pelvis (1.7%).

**Table 3:** Cause of injury by body region of 521 injury diagnoses in admitted patients with earthquake-related injuries.

Cause of Injury	Body Region								Total Number (%)
	Head	Face	Thorax	Spine	Upper Extremities	Lower Extremities and Pelvis	Abdomen	Body Surface and Others	
Hit/trapped by objects	39	11	18	13	6	44	1	44	176 (33.8)
Motor vehicle	1	3	1	0	1	8	0	3	17 (3.3)
Burns/scalds	0	0	2	0	0	6	0	1	9 (1.7)
Fall/slip	7	4	5	11	11	24	1	10	73 (14.0)
Cutting/piercing	0	0	0	0	11	1	0	1	13 (2.5)
Injured during rescue	3	0	5	2	6	8	0	8	32 (6.1)
Others	0	0	0	1	0	0	0	0	1 (0.2)

**Table 4:** Injury severity by body region of 521 injury diagnoses in admitted patients with earthquake-related injuries.

AIS Score	Body Region								Total Number (%)
	Head	Face	Thorax	Spine	Upper Extremities	Lower Extremities and Pelvis	Abdomen	Surface Area $ Others	
									
1 (minor)	44	18	9	1	23	60	2	83	240 (46.1)
3	7	2	18	3	5	29	0	1	65 (12.5)
4	3	0	1	1	0	0	1	0	6 (1.2)
5	2	0	0	2	0	3	0	0	7 (1.3)
6 (fatal)	0	0	0	0	0	0	0	0	0 (0)

A two-level spatial clustering was detected among the patients presenting with two injury diagnoses (Table 5). First, a patient presenting with contusion (laceration) and soft tissue injury was the prominent profile of this group (35.6%). In this group, head and body surface (16.9%), lower extremities and body surface (6.5%) and Upper extremities (3.9%) were the top three frequency anatomical regions occurred in the patients observed. Otherwise, 19.2% of the patients were diagnosed with the combination of fracture and soft tissue injury, most of which were focused on the spine & body surface (6.5%) and spine & lower extremities (3.9%). Fractures in neighboring anatomical sites gained importance in the patients with two fractures, most of which occurred in the same location, such as lower extremities (9.1%) and spine (2.6%). Other combinations of injury diagnoses by body region in two-injury patients were also shown in Table 5.

A total of 266 inpatients received 361 surgical procedures, with 1.3 procedures per patient. The most common procedure was sutures and dressings (33.7%), followed by open reduction and internal fixation (21.9%), and simple plaster application (15.8%).

**Table 5:** Most frequent combinations of injury diagnoses by body region in 73 two-injury patients.

Associated Injury Diagnoses	Number (%)
**Contusion & Laceration, Soft Tissue Injury**	**26 (35.6)**
Head, body surface	13
Lower extremities, body surface	5 (6.5)
Upper extremities	3 (3.9)
Head, lower extremities	2 (2.6)
Head, upper extremities	1 (1.3)
Head, Abdomen	1 (1.3)
Upper extremities, body surface	1 (1.3)
**Fracture, Soft Tissue Injury**	**14 (19.2)**
Spine, body surface	5 (6.5)
Spine, lower extremities	3 (3.9)
Lower extremities	1 (1.3)
Upper extremities	1 (1.3)
Lower & upper extremities	1 (1.3)
Lower extremities, head	1 (1.3)
Lower extremities, body surface	1 (1.3)
Rips, body surface	1 (1.3)
**Two Fractures**	**11 (15.1)**
Lower extremities	7 (9.1)
Spine	2 (2.6)
Lower extremities, face	1 (1.3)
Lower & upper extremities	1 (1.3)
**Two Contusion & Laceration Injuries**	**6 (8.2)**
Upper extremities	1 (1.3)
Head	1 (1.3)
Face	1 (1.3)
Head, face	1 (1.3)
Head, Lower extremities	1 (1.3)
Head, Thorax	1 (1.3)
**Fracture, Contusion & Laceration**	**4 (5.4)**
Lower extremities	3 (3.9)
Lower extremities, face	1 (1.3)
**Fracture, Dislocation**	**4 (5.4)**
Lower extremities	3 (3.9)
Upper extremities	1 (1.3)
**Closed injury, Soft Tissue Injury**	**3 (4.1)**
Head, body surface	2 (2.6)
Thorax, body surface	1 (1.3)
**Fracture, Closed Injury**	**2 (2.7)**
Thorax	2 (2.6)
**Fracture, Closed Injury**	**1 (1.3)**
Lower extremities	1 (1.3)
**Two Soft Tissue Injuries**	**1 (1.3)**
Thorax, lower extremities	1 (1.3)
**Dislocation, Soft Tissue Injury**	**1 (1.3)**
Spine, body surface	1 (1.3)

## 4. Discussion

Earthquakes are thought to be one of the most catastrophic natural disasters, which are characterized by a huge number of casualties during a very short period of time; they result in massive destruction of a country’s infrastructure, including medical facilities, which results in a shortage of medical and surgical treatment capabilities [12,13,14]. Variation in the number of earthquake deaths can be attributed to many categories of risk factors, such as geophysical factors, building factors, individual human characteristics related to the ability to respond to the earthquake, and the efficiency and organization of the rescue relief [15]. In the five years following the 2008 Wenchuan earthquake, the Yushu and Lushan earthquakes occurred in succession, within a similar area (western China), a similar period of time (14 April *vs.* 20 April), a similar magnitude scale (7.1 *vs.* 7.0 on the Richter scale), a similar focal depth (14 and 13 kilometers), a similar Max-intensity scale (9.0 degrees), and even a similar number of injuries (12,135 *vs.* 12,211). However, the death toll of the two earthquakes varied widely from each other (2698 *vs.* 196). To understand this interesting phenomenon, it is necessary to compare these two earthquakes in depth to explore the differences in the organization of the rescue efforts. Has significant progress been made since the Yushu earthquake? Is this reflected in the Lushan disaster relief? What are the differences in the injury profiles between Lushan and the 2010 Yushu earthquake and other similar disasters? Furthermore, it is also essential for us to share the experiences and lessons from the medical aid operations so that future disasters can be dealt with more effectively.

There is no doubt that initial decision to medically evacuate severely injured patients to nearby hospitals in the early days has perhaps been the most important aspect of medical aid operations in decreasing mortality and morbidity, especially when medical care facilities have been destroyed or severely damaged by an earthquake [16,17]. In the 2010 Yushu earthquake relief operation, air evacuation played a much more important role in the process of transferring injured victims and it ensured most of the injured were admitted to peripheral hospitals within the first three days. There are some factors that may account for this: (1) the Yushu prefecture is located in a remote, high-altitude region and roads to the epicentral area were seriously damaged [18], thus creating enormous difficulty for land evacuation; (2) the nearest city (Changdu) lies almost 490 km away from the Yushu prefecture, and therefore, land evacuation was much more time-consuming and inefficient; and (3) the Batang airport which was only 18 km away from the Yushu prefecture had been put into use in August 2009; fortunately, this allowed air transportation to provide timely assistance [19]. However, in the Lushan earthquake, land transportation, such as ambulances, private cars, buses, etc. were the primary source of transportation. Helicopter transport was also used, but only for those severely injured who could be transferred directly to the General Hospital of the Chengdu Military Region. Three reasons accounted for this: (1) there was no adjacent airport around the hard-hit regions, which added difficulty in landing the large fixed wing aircraft, (2) there were only a few suitable ground areas large enough to land helicopters, and (3) there were not enough helicopters to provide transport for all of the injured earthquake victims, which was a serious problem that restricted the scale of air evacuations. Fortunately, the roads were not seriously damaged, and 80.08% (*n* = 213) of the patients were admitted to the three military hospitals in the first two days; almost all of the victims were transported by land. Previous studies have demonstrated the merits of medical airlift for injuries, as this could lead to a 21% to 52% decrease in mortality, especially for traumatized patients [20,21]. This indicated a substantial need for specialized medical evacuation aircraft and resources for the transportation of those severely injured in the initial days after disasters. More attention should also be paid to the formal training of medical workers.

The earthquake-related injuries were mostly caused by buildings collapsing and being struck by objects; these accounted for 38.4% and 33.8% of the injuries, respectively, which was in accordance with previous studies [22]. It should be emphasized that 11 patients had injuries caused by slipping/falling, especially spine injuries, accounting for 4.1%. Interestingly, eight of them were injured and admitted in Chengdu city, the capital of Sichuan province, which is nearly 120 km from the earthquake epicenter and was affected very little by the Lushan earthquake. After a more in-depth investigation, we found that all of them jumped from buildings or took other dangerous ways to escape, which could cause unnecessary injuries when aftershocks occurred. Fortunately, they only suffered from bone fractures and there was no threat to their lives. Lessons should be learnt from these rather unusual events and more careful consideration should be given to instructing people on the correct methods and skills required for self-rescue during natural disasters. Further research is also required to determine suitable interventions for improving the mental health conditions of survivors in areas affected by earthquakes. Due to the economy and local lifestyle, stoves were popularly used for heating and cooking in the Lushan areas, and this might have been the major reason for nine patients (1.7%) sustaining burns and scalds. In 1995 following the Hanshin-Awaji earthquake, hospitalized patients with burns accounted for 1.9% of the injured victims [23]. Previous studies also indicated that earthquakes occurring in non-industrialized towns in the evening or at cooking times will result in burn injuries [24].

Although our findings were in general agreement with other studies reporting injury epidemiology after earthquakes [16,21,22,23,24], we report higher incidence of orthopedic injuries, particularly extremity fractures of the lower limbs. The most frequently injured anatomic sites were the lower extremities and pelvis (34.2%), followed by the body surface (17.9%), head (14.0%), and spine (10%). The lower extremities and pelvis were known as the highest risk location. In the present study, fractures accounted for 41.5% of the injuries; lower extremities and pelvis fracture accounted for 57.3% of the lower extremities and pelvis injuries, and spinal fractures accounted for 96.2% of the spinal injuries, which was similar to the injuries found after most of the other earthquakes studied [16,25,26,27,28]. It has been demonstrated that the incidence of fractures and injuries in an earthquake depends on the body posture and living status of the patients at the time of disasters [29]. If the patients are standing or sitting at the time of injury, the most frequent observed fractures will be those of the vertebral column and if the patients are lying in supine or lateral positions at the time of the earthquake, most of the fractures will be those of the pelvis and thoracic cage skeleton [30]. Head injury and thoracic injury reached 14.0% and 8.6%, respectively, indicating that such kinds of injuries should not be neglected when we pay more attention to the management of limb and pelvis injuries.

Through our analyses we found that more than half of the injured (55.64%) arriving at hospitals had multiple injuries. The most frequent combination injury was contusion and laceration (soft tissue injury). It is noteworthy that only 30 (11.2%) patients presented with three injuries or more since those with more injuries may die due to the severity of multiple injuries. Medical rescue teams arriving at the site must be prepared to care for patients with multiple injuries who may be at increasing risk of sepsis and multi-organ failure.

Crush syndrome is one of the most common of all the injuries resulting from an earthquake. It has been reported that there were 372 (6.09%) cases of crush syndrome following the 1995 Hanshin-Awaji earthquake [31]. The incidence rate was 33% among hospitalized patients following the 1999 Marmara earthquake [25]. In the present study, nevertheless, only six patients (1.2%) were identified as having significant crush syndrome. The remarkable difference may be due primarily to the type of building construction and materials. We found that traditional mud or adobe construction was popular in Lushan earthquake areas. Although poor seismic resistance caused many houses to be destroyed, victims in this type of construction were easier to rescue or able to rescue themselves soon after the disaster. Similar effects were observed in the Nicaragua earthquake [32], and the Guatemala earthquake [33], whereas a greater number of crush syndrome victims were diagnosed in the collapse of multistory buildings [34]. This may be one major reason for the low incidence of crush syndrome in our study.

While many engineering studies have almost always estimated deaths and injuries based on a correlation with building structural damage [35,36], there is evidence that the picture is considerably more complex. The damage to building and injuries to its inhabitants can greatly vary depending on its foundations, the underlying soil condition, building construction type, quality and year of construction, local surroundings, and even the occupant’s response during the earthquake, etc. However, it is axiomatic that more damage is associated with more fatalities. Data from the prevalent reinforced concrete frame buildings in Turkey suggests that the very small proportion of buildings that suffered pancake collapse were responsible for the vast majority of fatalities [35]. Fatality rates in heavily damaged buildings were 1.5 per 100 by comparison with 10.7 per 100 in totally collapsed buildings [36]. It has been proved that as far as fatality prevention, the most effective preventive effort would have been appropriate structural approaches prior to the earthquake [37].

In the present study, single injuries with an AIS score of 1–2 accounted for 86.1% of the injuries, indicating that most of the injuries were mild. One reason might be that some nonprofessional rescuers lacked experience or the skills of medical rescue during the early stages of the disaster, which could lead to the deaths of some severely injured patients, especially those with crush syndrome [16]. The other reason might be that the slightly injured victims were easier for the rescue team members to find and thus, they received timely treatment.

Our results showed that surgical procedures are the main treatment for earthquake injuries, including sutures and dressings (33.7%), followed by open reduction and internal fixation (21.9%) and simple plaster application (15.8%). Soft tissue injuries were often treated at the disaster site by medical rescuers with simple first aid techniques to prevent unnecessary overcrowding of hospitals. However, we still found a number of soft tissue injuries being treated at the hospitals. The reasons can be explained as follows: (1) the individual delay in seeking health care that led to complications; (2) the long commuting distances that made travel for most patients both logistically and monetarily less feasible for daily dressings [38]. In consideration of the fractures, the fixation is one of the most important operations. Previous studies advocated that early fixation could significantly decrease the incidence of pulmonary complications and organ failure to increase the survival rate [39].However, early definitive treatment for multiple injury patients may have side effects, indicating that the best time of operation for multiple injury patients is 2–5 days after injury [40,41]. In our study, however, most were treated with closed reduction and external fixation (29.7%) at the early stage due to the limitations of their medical condition.

The current study has a few limitations that are worth noting. In our study, most epidemiologic data of earthquake patients rely on the medical records of surveyed hospitals since the casualties in quake-hit regions were difficult to evaluate during chaotic disaster conditions. However, detailed medical records of all hospitalized patients were often incomplete because of the initial disorganization, especially during the first few hours after the earthquake [25]. Besides，the classification of the patients’ injuries was based on the diagnosis after hospitalization or at discharge, which means the injury information from the emergency department was unavailable in our study.

## 5. Conclusions

A catastrophic earthquake hit Sichuan province since the 2008 Wenchuan and the 2010 Yushu earthquakes, which could be considered a touchstone to reflect on the progress of the emergency rescue capability of China. Unlike the Yushu earthquake, land transportation for severe injuries played a crucial role in decrease the mortality and morbidity. It is necessary for hospitals to initiate effective emergency measures while facing the peak admission flow within the initial 48 h period. Earthquake-related injuries were mostly caused by buildings collapsing and victims being struck by objects; the most frequently injured anatomic sites were the lower extremities and pelvis, body surface, and head. Fractures were the most frequent injury but crush syndrome was relatively low due to the special housing structures in the Lushan area. More attention should be paid to the importance of instructing correct methods of self-rescue to avoid unnecessary injuries when aftershock occurs. It is essential for us to share these experiences and lessons from our medical aid operations so that future disasters can be dealt with more effectively.

## Key Messages

- Emergency transportation for severe injuries in the early phase after the earthquakes played a crucial role in decrease the mortality and morbidity.
- It is necessary for hospitals to initiate effective emergency measures while facing the peak admission flow within the initial 48-hour period.
- The injuries caused by earthquakes were always complex and of various patterns, which presented sudden and serious crisis for health management and treatment centers
- More attention should be paid to the importance of instructing correct methods of self-rescue to avoid unnecessary injuries when aftershock occurs.
